# Postpartum spontaneous coronary, vertebral, and mesenteric artery dissections: a case report

**DOI:** 10.1186/s13256-016-0937-0

**Published:** 2016-06-08

**Authors:** Sean Spence, Maneesh Sud, Ravi Bajaj, Anna Zavodni, Sharron Sandhu, Mina Madan

**Affiliations:** 1 Division of Cardiology, Department of Medicine, Sunnybrook Health Sciences Centre, University of Toronto, Toronto, Ontario Canada; 2 Division of Rheumatology, Department of Medicine, Sunnybrook Health Sciences Centre, University of Toronto, Toronto, Ontario Canada; 3 Department of Medical Imaging, Sunnybrook Health Sciences Centre, University of Toronto, Toronto, Ontario Canada; 4 Sunnybrook Health Sciences Centre, 2075 Bayview Ave., Room D3 80, Toronto, Ontario M4N 3M5 Canada

**Keywords:** Coronary artery dissection, Acute myocardial infarction, Vertebral artery dissection, Gender

## Abstract

**Background:**

Spontaneous coronary artery dissection is a rare cause of myocardial infarction that must always be considered on a clinician’s differential diagnosis, particularly in patients <50-years old with a paucity of typical vascular risk factors.

**Case presentation:**

We describe a case of a 33-year-old white woman, 3 weeks postpartum, presenting with retrosternal chest and back pain, neck pain and stiffness, and intermittent headaches. Subsequent workup revealed concurrent spontaneous dissections in three separate medium-sized arterial beds.

**Conclusions:**

She was successfully managed in a conservative fashion, highlighting that percutaneous or surgical revascularization can often be foregone in favor of conservative medical therapy.

## Background

Spontaneous coronary artery dissection (SCAD) is a rare cause of acute myocardial infarction (MI). It is being increasingly recognized as a non-atherosclerotic etiology for MI in patients <50-years old, predominantly women <50-years old [1]. SCAD is currently thought to have a complex multifactorial etiology with associations to atherosclerosis, the peripartum period, multiparity, menopause, oral contraceptive use, connective tissue disease, trauma, psychophysical stress, vascular malformations, cocaine use, vasculitis, fibromuscular dysplasia, polycystic kidney disease, and certain medications [2]. Although three case reports have been published for concurrent spontaneous coronary and vertebral artery dissections (VADs), we present, to the best of our knowledge, the first case of postpartum multivessel coronary, cerebral, and mesenteric artery dissection [3–5]. The only risk factor present in these other cases was the postpartum state, and two of the three patients presented with symptoms of headache and angina. With this unusual case we highlight the importance of a systematic approach to diagnosis and management, once SCAD is suspected.

## Case presentation

A 33-year-old white woman (G_2_P_2_), with previously repaired sinus venosus atrial septal defect (ASD), underwent emergency coronary angiography for an ST-elevation myocardial infarction (STEMI) 3 weeks postpartum. She had no traditional risk factors for coronary artery disease, both her pregnancies were uneventful, and there was no significant family history or past medical history aside from the aforementioned ASD. She endorsed a 1 to 2-week history of intermittent nonexertional chest, back, and neck pain, neck stiffness, and headaches. On the day of admission, she presented to another hospital with severe persistent chest and back pain. Her presenting electrocardiogram (ECG) showed a sinus rhythm with anterior and inferior ST-segment elevation and reciprocal ST-segment depression in the lateral leads.

**Fig. 1:**
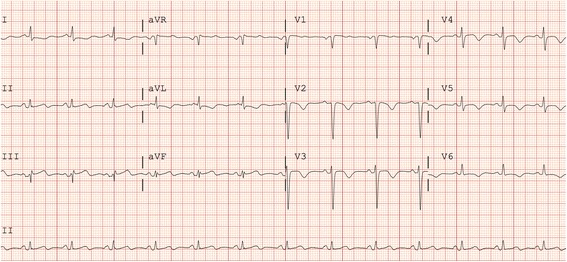
Electrocardiogram on arrival to our cardiac catheterization laboratory showing resolving inferior ST elevation and T wave inversion across the precordial leads

Her creatine kinase was 1018 U/L and high-sensitivity troponin T was 1828 ng/L. Upon arrival at our cardiac catheterization laboratory, her chest discomfort was resolving, and a repeat ECG showed slight persistence of ST elevation inferiorly, and T wave inversions across the precordial leads (Fig. 1).

**Fig. 2:**
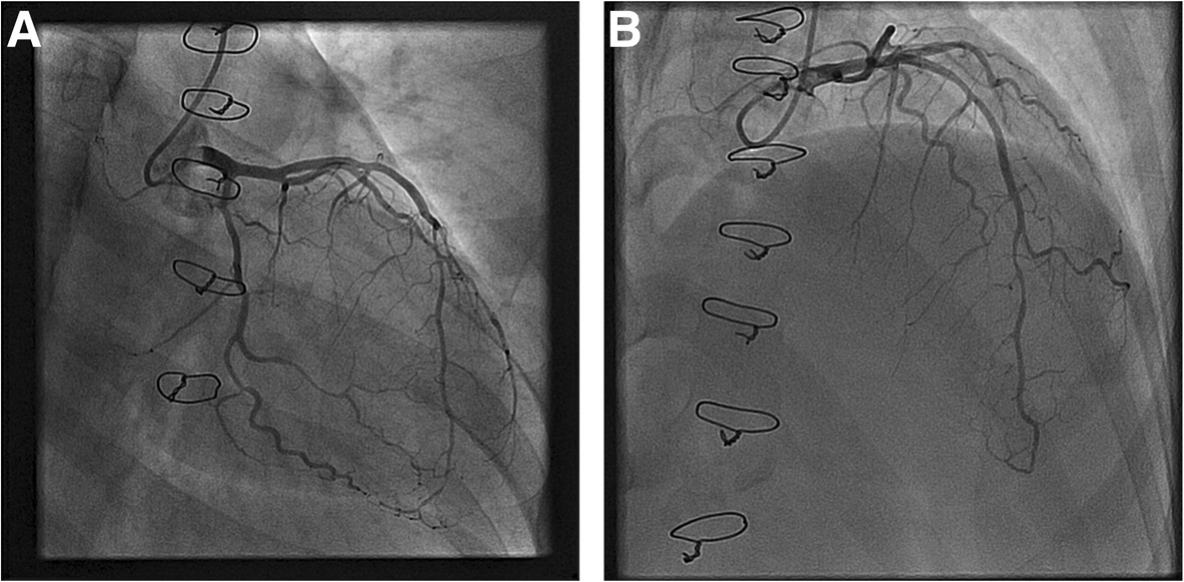
Selective coronary angiography showing abrupt tapering of the second obtuse marginal branch of the left circumflex artery (**a**) and mid-left anterior descending artery (**b**)

She was noted to be hypertensive at 145/99 (equal in both arms). Immediate bedside echocardiography demonstrated normal aortic root dimensions, and absence of ascending aortic dissection or aortic insufficiency. Her overall left ventricular (LV) function was preserved. Coronary angiography revealed normal right coronary and left main arteries. There was abrupt tapering with significant stenosis noted in her mid-left anterior descending (LAD) artery and the second obtuse marginal branch (OM2) of her left circumflex artery consistent with SCADs (Fig. 2).

Because Thrombolysis In Myocardial Infarction (TIMI) grade 3 flow was present in all vessels, coronary intervention was deferred and our patient was managed conservatively.

**Fig. 3:**
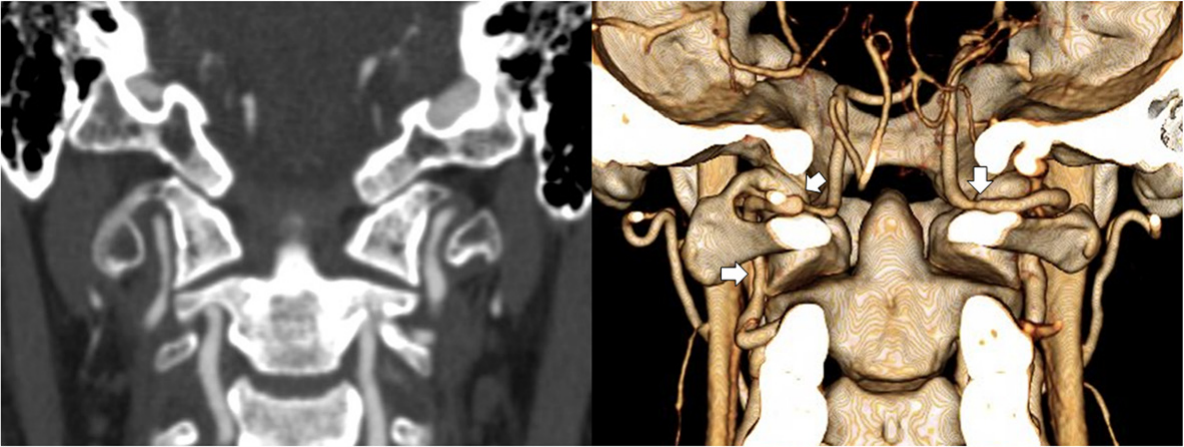
Contrast-enhanced computed tomography angiogram of the head and neck demonstrates bilateral vertebral artery dissections. A coronal reformat (*left panel*) and three-dimensional reconstruction (*right panel*), illustrate that both vertebral arteries are significantly narrowed (*arrows*) along a long irregular segment that extends from the C1–C2 level to the dural penetration point. The three-dimensional reformat best demonstrates the formation of multiple small pseudoaneurysms along the course of the affected segments

At 24 hours, echocardiography revealed grade 3 LV systolic function with severe hypokinesis of the inferior, posterior, and apical regions, without LV thrombus. Cerebral computed tomography angiography (CTA) revealed bilateral sub-acute VADs (Fig. 3).

**Fig. 4:**
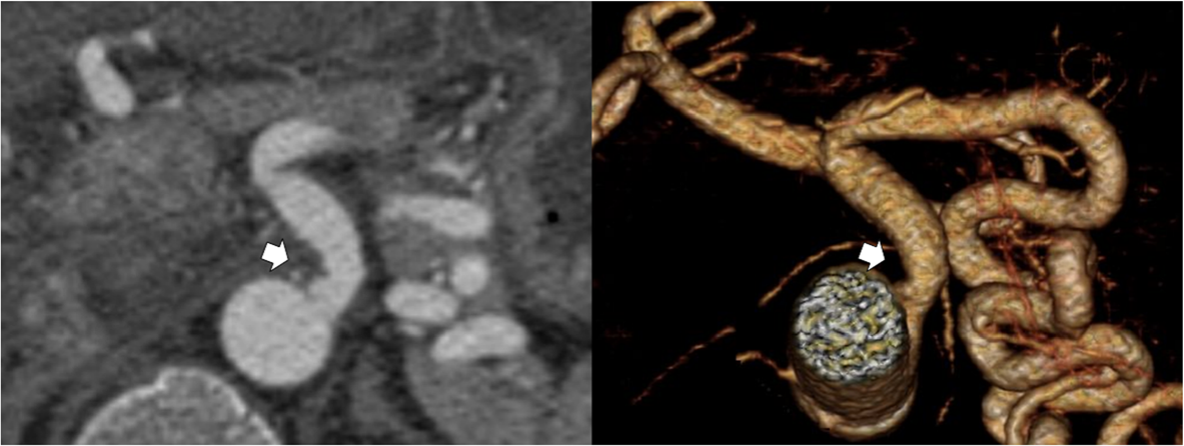
Contrast-enhanced computed tomography angiogram of the abdomen demonstrates a dissection of the celiac trunk (*arrows*). Axial (*left panel*) and three-dimensional (*right panel*) reconstruction images show an abrupt narrowing of the vessel in the absence of atherosclerotic plaque or extrinsic compression

Follow-up thoracic and abdominal CTA, to assess for fibromuscular dysplasia, connective tissue disease-related aortopathy or vasculitis, demonstrated normal renal arteries without aortopathy. However, dissections of her celiac trunk, superior mesenteric artery, and inferior mesenteric artery were noted (Fig. 4). Screening serologies including C-reactive protein (CRP), erythrocyte sedimentation rate (ESR), antinuclear antibodies (ANA), perinuclear antineutrophil cytoplasmic antibody (p-ANCA), cytoplasmic antineutrophil cytoplasmic antibody (c-ANCA), C3, and C4 levels were negative. Testing for a panel of genes associated with familial aortic aneurysms and dissections was negative. She remained clinically stable throughout her hospitalization without evidence of new ischemic symptoms. She was discharged on aspirin, clopidogrel, atorvastatin, metoprolol, ramipril, amlodipine, and transdermal nitroglycerin.

At 3 months follow-up, contrast-enhanced magnetic resonance angiography demonstrated resolution of her VADs and no residual luminal irregularities. Furthermore, at 15 months, an abdominal CTA did not reveal any dissections within her major aortic branches and coronary angiography revealed complete healing of her LAD and OM2 without residual stenosis. Selective renal angiography at the time of coronary angiography demonstrated mild beading of her right renal artery, and a normal left renal artery. Despite the non-diagnostic abdominal CTA, the changes noted on renal angiography were suggestive of an underlying diagnosis of fibromuscular dysplasia (FMD) with her clinical events possibly linked to the postpartum period. Clopidogrel was discontinued at 15 months with no further dissection events by 18 months of follow-up.

## Discussion

SCAD is an uncommon presentation of coronary artery disease accounting for 0.1 to 4.0 % of all acute coronary syndromes (ACSs) [1]. Patients are typically young, female, and lacking traditional coronary risk factors [1]. STEMI occurs in 48 % of cases, which are subclassified according to associations with known atherosclerotic disease, an absence of known coronary risk factors, and the peripartum period [2]. The pathophysiology and etiology of SCAD is poorly understood and felt to be multifactorial in etiology [1, 2].

The management of SCAD lacks consensus as a result of low incidence, heterogenous presentation, and lack of observational or clinical trial data. Registry studies do exist but the number of cases is low and there is little data to support specific management approaches. Treatment is largely guided by extrapolation of usual STEMI care and clinical experience. Initiation of dual antiplatelet therapy ACS is common prior to the diagnosis of SCAD and is typically continued for 12 months afterwards [1]. Thereafter, aspirin or clopidogrel monotherapy is usually sustained indefinitely. Although dual antiplatelet therapy appears to be associated with angiographic resolution of SCAD in many published cases, the ideal duration of therapy is unknown [6]. Similarly, the evidence for beta-blockade in SCAD is limited [6]. Its use is supported by a reduction in shear stress on vessel walls observed in populations with arteriopathies such as vascular Ehlers–Danlos syndrome and Marfan syndrome as well as in non-syndromic aortic dissection [1, 7].

Revascularization is typically considered in cases refractory to conservative management. Coronary stenting has been employed in several cases, albeit with some risk of extension of dissections during stent implantation. Coronary artery bypass grafting is generally reserved for cases with dissection of the left main or multiple vessels. Anticoagulation must be considered cautiously, as the benefits of preventing intracoronary thrombus formation must be weighed against the risks of worsening expansion of coronary intramural hematoma.

VAD is nearly four times more common than SCAD, with a reported incidence of 0.97/100,000 [8]. Not uncommonly, it accompanies SCAD, particularly in the context of connective tissue disease [9]. Consequently, a low threshold to investigate for extracoronary dissection in SCAD should exist, particularly given the potential to diagnose an associated connective tissue disease with its management implications. In patients with SCAD, even the smallest headache should not go uninvestigated. Antithrombotic therapy, utilizing either an anticoagulant or an antiplatelet agent, is the mainstay of therapy for ischemic stroke or transient ischemic attack (TIA) resultant from carotid artery dissection or VAD. Duration of therapy is dictated by the degree of vessel healing on repeat imaging at 3 and 6 months. Anticoagulation is typically avoided in intracranial dissections for fear of causing subarachnoid hemorrhage.

Although case reports have been published describing concurrent SCADs and VADs, we present, to the best of our knowledge, the first case of concurrent postpartum multivessel coronary, cerebral, and mesenteric artery dissections most likely in the background of an underlying diagnosis of FMD [3–5]. Although a well-designed cohort study has found a high prevalence of non-coronary FMD among patients with SCAD (86 %), only 12 % of patients had dissections of >1 coronary artery, and none were reported to have concomitant dissections in a non-coronary vascular bed [10]. Patients in this study were chiefly young women (98 % female, average age 51), and MI occurred in all cases: 30 % STEMI, 70 % non-ST-elevation myocardial infarction (NSTEMI). Unsurprisingly, there was a paucity of classic vascular risk factor among these patients, with only 4 % having diabetes, 20 % having dyslipidemia, and 30 % having hypertension. Only 1 of the 50 cases was associated with the postpartum period, and emotional stress was identified as a factor in 26 % of cases.

Of the three previously published cases of concurrent VAD and SCAD, one was managed with coronary stenting and medical therapy for VAD, the second was managed with coronary bypass surgery and medical therapy for VAD, and the third was managed with medication alone. Medical management was itself heterogeneous among these cases. Given the locations of the coronary dissections in our case, we opted for conservative management utilizing dual antiplatelet therapy, statins, beta blockade, and afterload reduction. Response to therapy was sufficient such that surgical or percutaneous coronary interventions were unnecessary. Given the lack of ischemic symptoms from the vertebral and intra-abdominal arterial dissections, in addition to the intradural extension of one of the VADs, anticoagulation was not employed.

This unusual case involves concurrent spontaneous dissections in three separate medium-sized arterial beds. It is also the first reported case successfully treated with the conservative measures described above and it underscores the importance of considering uncommon etiologies in individuals with atypical cardiac presentations. Once a diagnosis of SCAD is suspected, this case illustrates the importance of systematic evaluation for involvement of other vascular territories when compatible symptoms arise.

## Conclusions

Here we report a case of concurrent spontaneous dissections involving the coronary, vertebral, and mesenteric arterial beds in a 33-year-old woman who was 3 weeks postpartum and a presumptive diagnosis of FMD. She was successfully managed in a conservative fashion, without the need for percutaneous or surgical revascularization. Once a diagnosis of SCAD is suspected, this case highlights the importance of systematic medical imaging to determine the involvement of other vascular territories. Long-term observational registries are needed to understand the natural history of this condition.

## Abbreviations

ACS, acute coronary syndrome; ASD, atrial septal defect; CTA, computed tomography angiography; ECG, electrocardiogram; FMD, fibromuscular dysplasia; LAD, left anterior descending; LV, left ventricular; MI, myocardial infarction; OM2, second obtuse marginal branch; SCAD, spontaneous coronary artery dissection; STEMI, ST-elevation myocardial infarction; VAD, vertebral artery dissection.
